# Hydrogel-based scaffolds to support intrathecal stem cell transplantation as a gateway to the spinal cord: clinical needs, biomaterials, and imaging technologies

**DOI:** 10.1038/s41536-018-0046-3

**Published:** 2018-04-04

**Authors:** J. Miguel Oliveira, Luisa Carvalho, Joana Silva-Correia, Sílvia Vieira, Malgorzata Majchrzak, Barbara Lukomska, Luiza Stanaszek, Paulina Strymecka, Izabela Malysz-Cymborska, Dominika Golubczyk, Lukasz Kalkowski, Rui L. Reis, Miroslaw Janowski, Piotr Walczak

**Affiliations:** 1 3B´s Research Group – Biomaterials, Biodegradables and Biomimetics, University of Minho, Headquarters of the European Institute of Excellence, Tissue Engineering and Regenerative Medicine, AvePark, Zona Industrial da Gandra, 4805-017 Barco, Guimarães Portugal; 2 ICVS/3B’s - PT Government Associate Laboratory, Braga, Portugal; 3 The Discoveries Centre for Regenerative and Precision Medicine, Headquarters at University of Minho, Avepark, 4805-017 Barco, Guimarães Portugal; 4 NeuroRepair Department, Mossakowski Medical Research Centre, Polish Academy of Sciences, Warsaw, Poland; 5 Department of Neurology and Neurosurgery, School of Medicine, Collegium Medicum, University of Warmia and Mazury, Olsztyn, Poland; 6 Russel H, Morgan Department of Radiology and Radiological Science, Johns Hopkins University, Baltimore, MD USA; 7 Vascular Biology Program, Institute for Cell Engineering, Johns Hopkins University, Baltimore, MD USA

## Abstract

The prospects for cell replacement in spinal cord diseases are impeded by inefficient stem cell delivery. The deep location of the spinal cord and complex surgical access, as well as densely packed vital structures, question the feasibility of the widespread use of multiple spinal cord punctures to inject stem cells. Disorders characterized by disseminated pathology are particularly appealing for the distribution of cells globally throughout the spinal cord in a minimally invasive fashion. The intrathecal space, with access to a relatively large surface area along the spinal cord, is an attractive route for global stem cell delivery, and, indeed, is highly promising, but the success of this approach relies on the ability of cells (1) to survive in the cerebrospinal fluid (CSF), (2) to adhere to the spinal cord surface, and (3) to migrate, ultimately, into the parenchyma. Intrathecal infusion of cell suspension, however, has been insufficient and we postulate that embedding transplanted cells within hydrogel scaffolds will facilitate reaching these goals. In this review, we focus on practical considerations that render the intrathecal approach clinically viable, and then discuss the characteristics of various biomaterials that are suitable to serve as scaffolds. We also propose strategies to modulate the local microenvironment with nanoparticle carriers to improve the functionality of cellular grafts. Finally, we provide an overview of imaging modalities for in vivo monitoring and characterization of biomaterials and stem cells. This comprehensive review should serve as a guide for those planning preclinical and clinical studies on intrathecal stem cell transplantation.

## Introduction

Central nervous system (CNS) diseases and injuries are some of the most devastating for patients. The complexity and role of the CNS is such that its functional deterioration results in a huge impact on the quality of life, as well as an enormous financial burden to society. Cellular degeneration and death are the most common features of CNS disorders. In that way, several approaches that have attempted to regenerate cells, tissues, or organs in order to restore or establish normal function have been studied. In many instances, transplanted stem cell suspensions were shown to be highly therapeutic in small-animal models,^1^ but that was attributable to the broad distribution of transplanted cells in the CNS.^2^ The attempt to translate these exciting results to the clinical scenario has been challenging. While several clinical trials report therapeutic benefit,^3,4^ many other trials report good safety profile but no efficacy,^5–7^ triggering the closing of some cell-manufacturing companies. Such disappointing clinical translation results can be attributed to the large difference in the size of the CNS between mice and humans, as the mouse brain is 1000 times smaller. The issue of cell distribution in the large CNS must be addressed prior to the pursuit of more clinical research. Herein, we discuss the current clinical needs and solutions that have been used in cell-based therapies, with a particular focus on targeting the spinal cord. Recent reports dealing with hydrogels and nanoparticles for cell delivery to the CNS are also reviewed. The modulation of the microenvironment of cell-laden hydrogels with the use of nanoparticles and engineering strategies to allow in vivo imaging are also discussed in depth.

## Targeting the spinal cord: clinical needs and solutions

**Fig. 1:**
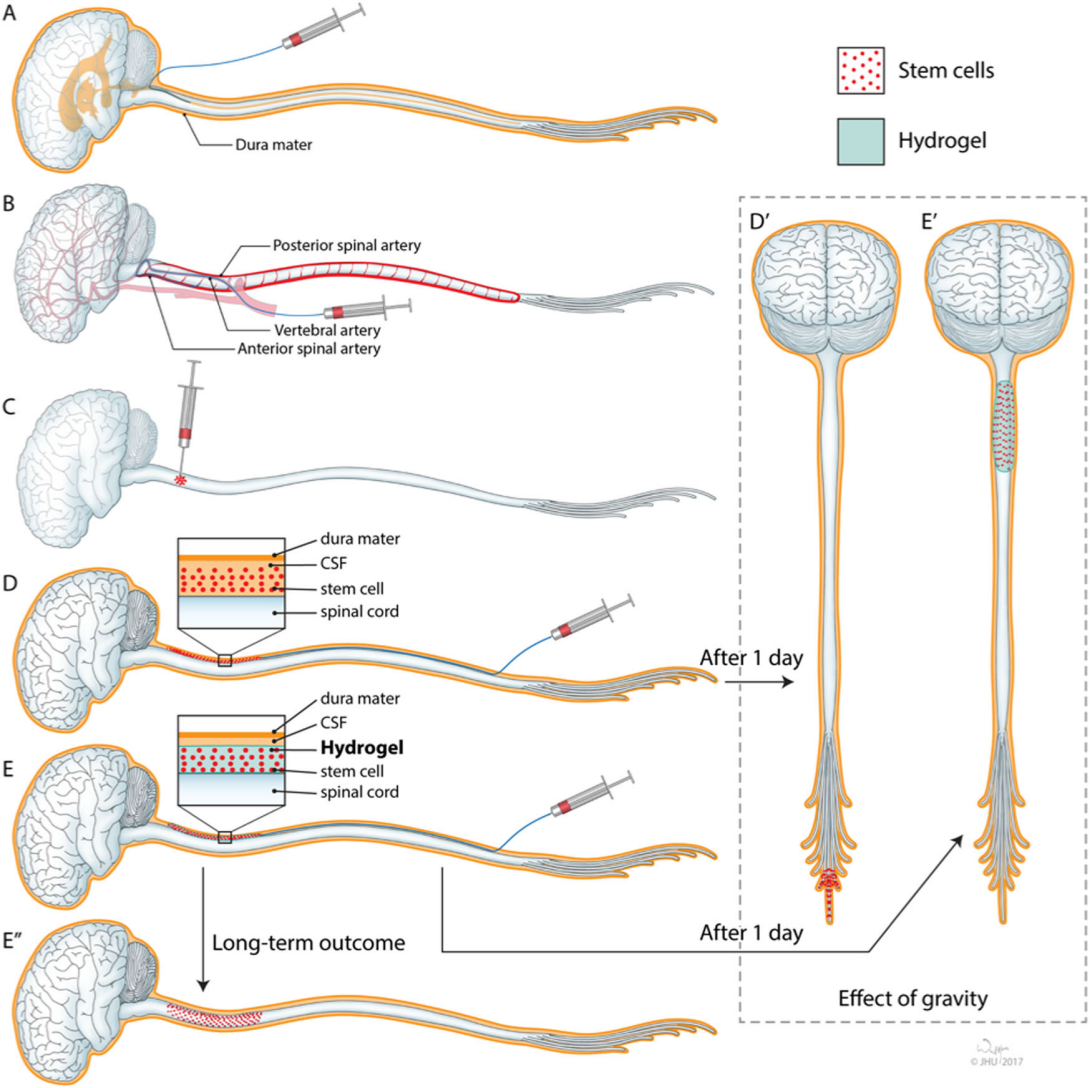
Injection routes of stem cell/biomaterial constructs into the spinal cord

Intraventricular^8^ and intra-arterial^9^ routes are very promising for the delivery of stem cells to the brain. However, efficient delivery of stem cells to the broad areas of the spinal cord needs still to being resolved. There are several gateways to the spinal cord that have been considered, including the central canal, the intra-arterial, the intraparenchymal, and/or the intrathecal routes. Schematic representation of the cell/biomaterial constructs delivery routes into the spinal cord is depicted in Fig. 1.

### Central canal

The central canal of the spinal cord, an extension of the ventricular system, is a relatively narrow space, which also plays a central role in the CSF circulation. The obstruction of the cerebrospinal fluid (CSF) circulation following injection of stem cells could lead to a very debilitating disorder, syringomyelia,^10^ and thus, this route of cell delivery should be pursued clinically only after extensive research on large animals (Fig. 1a).

### Intra-arterial

Blood for the spinal cord is supplied by a number of small segmental arteries, which are difficult to reach with an endovascular catheter, and, importantly, the obstruction of these arteries can result in serious and disabling consequences.^11^ Considering that most of the potential targets for therapy are within the cervical spine, any vascular occlusion or injury in this area may result in severe neurological deficits that could affect most of the body, including tetraplegia. In this context, the intra-arterial route for cell delivery to the spinal cord should be considered with extreme caution (Fig. 1b).

### Intraparenchymal

Direct needle injections, including multi-site injections, are currently the most actively pursued strategy with which to deliver stem cells to the spinal cord, as it has been shown to be effective in small animals.^12^ While the procedure has been shown to be safe in large animals^13,14^ and open-label phase I/II clinical trials,^15,16^ the puncture of the spinal cord is a very complex and expensive procedure. Intraparenchymal delivery is also not well suited for disorders with global or multifocal pathology, as each injection delivers cells to only a relatively small volume of tissue. Multiple injections might be considered to improve cell biodistribution; however, that amplifies the risk of damage and any potential therapeutic effects could be offset by the procedure-related morbidity. The need for neurosurgical virtuosity and specialized injectors might limit the widespread application of this route. The invasive nature of intraparenchymal injection requires the ability to gain surgical access to the spinal cord, including muscle dissections and multilevel laminectomies. In addition, repeated needle insertion increases the risk for injury of a parenchymal vessel, with the resultant neurological consequences. Therefore, this approach is another example where direct translation of therapeutic effectiveness from small animals to patients may be challenging (Fig. 1c).

The spinal cord is thus a difficult target in which to achieve effective, global delivery of stem cells and there is a clear clinical need for a robust, minimally invasive, and safe method for efficient and repeatable stem cell delivery to the spinal cord. The low volume-to-surface ratio (in contrast to the brain) and easy access speaks in favor of using the extraspinal space as a route of cell delivery.

### Intrathecal

The extraspinal space is filled with a CSF, and, most importantly, can be easily approached through a lumbar puncture, a method routinely used to sample the CSF, for drug delivery, as well as to lower CSF pressure during neurosurgical procedures. The use of widely available fluoroscopic guidance allows placement of a catheter tip at the desired position, such as the cervical area.^17^ Thus, if this route were effective, it could immediately be applied worldwide; however, several challenges need to be addressed before this approach can become a clinical reality. The most important is that the cells deployed to the CSF need to traverse the pia matter and enter the parenchyma of the spinal cord in the desired region. There is strong evidence about ability of the cells to migrate from the CSF compartments into parenchyma on early stage of development;^2^ however, adult or aging pia matter will likely be more challenging barrier to traverse. Some potential solutions to this challenge could be engineering the cells for expression of metalloproteinases^18^ or supplementation of biomaterials with enzymes selectively targeting collagen and reticular fibers of pia. The second challenge is that the cells injected as a suspension (Fig. 1d) settle due to gravity, and, in humans, usually accumulate below the spinal cord around the cauda equina (Fig. 1d’). Potentially, both challenges, i.e., adhesion to the spinal surface and sedimentation can be addressed by embedding cells within the hydrogel-based scaffold (Fig. 1e,e’,e”). This, in turn, calls for providing an appropriate environment that would match the requirements of the cell type and could be addressed by the use of nanoparticles that could slowly release the desired trophic factors. In subsequent sections, we will extensively review the options for the biomaterials (hydrogels and nanoparticles) to address the unmet need for stem cell delivery to the spinal cord via the intrathecal space.

## Hydrogels as scaffolds for stem cell delivery

The application of appropriate scaffolding biomaterial matrices has gained a new impetus to repair the CNS because of the huge developments in cell engineering and cell-based treatment solutions. Scaffolds, by including natural extracellular matrix (ECM) proteins, can direct cell behavior by providing cues about cells during migration, differentiation, and regeneration in the CNS environment.^19^ In particular, the use of hydrogel systems is an attractive approach for stem cell delivery, as these systems can serve as temporary mimetic niche with which to support the survival of transplanted cells or recruited endogenous cells at the lesion site to promote recovery.^20^ The high-water content and tissue-like mechanical properties of hydrogels make them highly attractive scaffolds for implantation in soft tissue.^21^ Furthermore, the hydrogels present porous structures that allow cell attachment and growth, as well as the “smart” release of biological agents at the injury site.^21,22^

With respect to application in the spinal cord, hydrogels are the system of choice to provide the appropriate mechanics for precise localization and spatio-temporal control of cell delivery, and that is particularly important in case of transplantation to the intrathecal space. The rationale for using injectable hydrogels for intrathecal stem cell delivery is based on the observations that stem cells transplanted into the CSF are subject to gravitational sedimentation.^8^ In addition, cells injected into the CSF may lack cell–cell contact and RGD signaling. Indeed, it has been shown that RGD signaling provided as part of the biomaterial system improves cell survival and function.^23^ The quality of hydrogels that is critical, with respect to their use in highly sensitive tissues of the CNS, is that they are biocompatible, minimizing adverse tissue reaction in vivo.^24^ Hydrogels can be easily tuned (composition and functionalization) in order to facilitate injectability. Their biomechanical properties can be adjusted for smooth flow with low resistance during infusion, as well as to broadly mimic the host microenvironment composition, including physical and mechanical characteristics representative of native CNS tissue, which minimizes mechanical mismatch.^25^

Both physical and covalently cross-linked hydrogels have been applied to the treatment of spinal cord injury (SCI). Until now, only a few studies reported the development of injectable hydrogel systems for the intrathecal delivery of cells to the CSF. Physical gels are simpler to use, since they typically do not involve harsh cross-linking conditions, thus reducing the possible toxicity normally associated with the use of covalent coupling agents.^26^ In fact, these types of gels often require a gelation-triggering system, such as local cooling,^22^ which introduces the risk of tissue damage, and, in case of intrathecal injection, is not applicable.

**Fig. 2:**
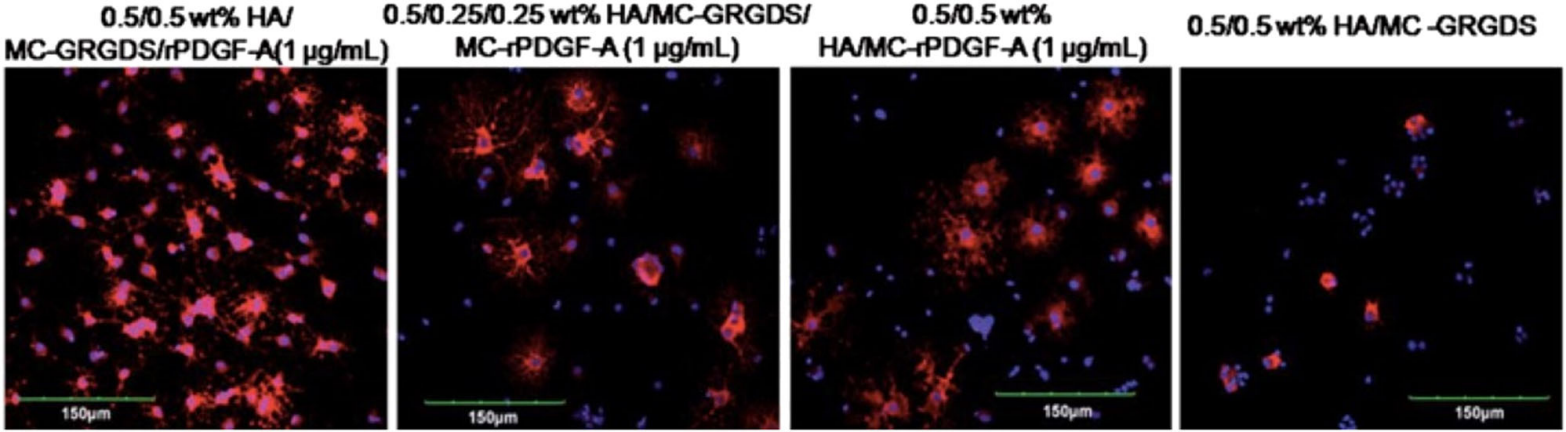
Effect of immobilization of GRGDS peptide on HAMC hydrogels for the differentiation of rat NSPCs. Confocal images of rat NSPCs after encapsulation in 0.5/0.5 wt% HAMC gels for 7 days. Cells were stained for anti-RIP (for oligodendrocytes, red) and counterstained with DAPI (for cell nuclei, blue). Reprinted from Tam et al.^30^

Chemically cross-linked hydrogels, however, are relevant because they present more desirable properties than physical gels in terms of flexibility to manipulate physical and biological properties. Synthetic functional groups can be incorporated in the polymers to enable (or enhance) gelation, for improved in vivo stability, or to enhance specific enzymatic susceptibility of the hydrogels.^27^ Chemically cross-linked hyaluronan is an example where researchers exploited the biocompatible, biodegradable, angiogenic, and anti-inflammatory nature of the base polymer. For example, Gupta et al.^28^ described, for the first time, the physical blend of an HA and methyl cellulose (HAMC)-injectable hydrogel to deliver NSCs into the rat brain after a clip compression injury. Also, Mothe et al.^29^ examined the survival and efficacy of adult brain-derived neural stem/progenitor cells (NSPCs) injected within a modified HAMC hydrogel with recombinant rat platelet-derived growth factor-A (rPDGF-A). The authors showed that, while only a limited number of surviving cells was found, the hydrogel promoted the survival of host neurons and oligodendrocytes, which was associated with better functional recovery on the ladder walk.^29^ Further, the same authors reported that the modification of the previously used HAMC with RGD promoted the survival, integration, and differentiation of human pluripotent stem cell-derived oligodendrocyte progenitor cells^30^ (Fig. 2). While over-proliferation of the cells was reported in that study, it demonstrated that the modified HAMC hydrogel reduces tumor formation by promoting differentiation in vivo. Control animals that received cells without hydrogel demonstrated extensive tumor formation and a decline in motor function.^31^ A combination of peptide-modified gellan gum (GG-GRGDS) hydrogels also showed a great potential to be used in cellular therapies designed to treat SCI. A co-culture of adipose stem cells (ASCs) and olfactory ensheathing cells (OECs) encapsulated into the GG-GRGDS hydrogel resulted in significant motor and histological improvements of SCI in rats.^32^

In brief, the selection of biomaterials, the surface morphology of the substrates, neurotrophic factors, cell density, and the effect of serum and many other factors can all affect the proliferation and differentiation of cultured neural stem cells. When envisioning the design of the cell culture environment in vitro and in vivo, as many factors as possible related to biomaterials should be considered and optimized to achieve the best biological performance, in the short-term, medium-term, and long-term. All these studies provide a promising strategy, combining a biocompatible and injectable polymer and stem cells to form an effective cell transplantation system for the treatment of spinal cord diseases. In order to develop more efficient treatment solutions, future research should focus on optimizing the conditions for augmenting stem cell survival, proliferation, migration, differentiation, and integration into the host tissue.

## Nanoparticles for the modulation of the microenvironment of cell-laden hydrogels

One of the most important characteristics of hydrogels that determine local microenvironment and injectability are their rheological properties^33^ and it has been shown that the rheological properties of a given hydrogel vary.^34^ When a hydrogel is injected, its rheological behavior and viscoelastic properties (storage modulus and loss modulus, G’ and G”, respectively) are negatively affected. In order to improve the viscoelastic properties, without altering the gel-like behavior of both acellular and cell-laden hydrogels, nanoparticles have been used as a reinforcement or filler material.^33,35^

In fact, the use of nanoparticle-based systems has expanded interest because of the exciting prospects.^36^ The use of materials at the nanoscale provides the extraordinary possibility to modify some of the properties of therapeutic carriers, such as solubility, diffusivity, biodistribution, release characteristics, and immunogenicity. In addition, these kinds of carriers show a longer circulation half-life, superior bioavailability, and lower toxicity.^37,38^

**Fig. 3:**
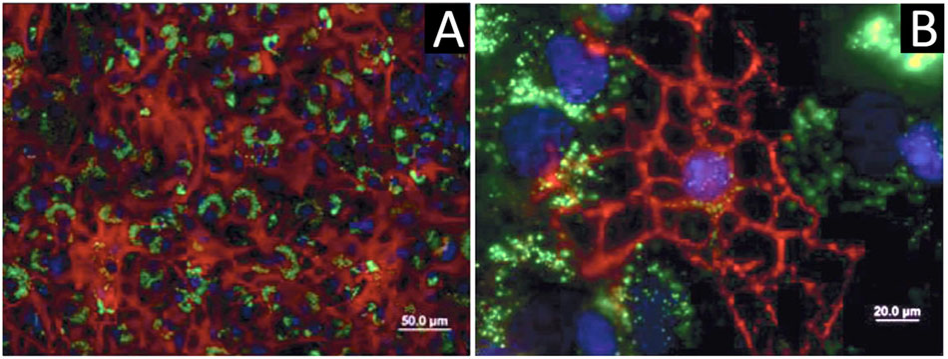
Internalization experiments within cortical glial cell cultures. **a** Astrocytes were able to internalize the FITC-labeled CMCht/PAMAM dendrimer nanoparticles after 48 h of incubation. **b** Oligodendrocytes also were able to internalize the FITC-labeled CMCht/PAMAM dendrimer nanoparticles. Representative image of the nanoparticles distributed along the intracellular compartment. Adapted from Salgado et al.^39^

Despite the large number of nanocarriers that are being developed and investigated for specific drug delivery purposes in the CNS, only a few studies have reported its application in the CSF. The vast majority of NPs for the CNS range from classical linear polymers to novel spherical molecules. For example, dendrimers that have a spherical morphology and highly branched have been shown to be interesting nanocarriers for CNS applications. They facilitate surface functionalization and control over their size, which influences the drug payload and targeting features.^39,40^ There are different dendrimers that have been exploited extensively for drug delivery, including poly(amidoamine) (PAMAM), poly(etherhydroxylamine) (PEHAM), and poly(propyleneimine) (PPI) dendrimers.^41^ Because of their unique structures and properties, PAMAM dendrimers have been the most investigated for the use in CNS applications. In fact, Oliveira et al. reported a new functionalization for a PAMAM dendrimer, by grafting it to a carboxymethylchitosan (CMCht) to improve the loading capacity.^42^ That nanocarriers were shown to be internalized by primary neurons and glial cells in culture system, suggesting their utility for CNS indications^39^ (Fig. 3). In another study, the corticosteroid, methylprednisolone (MP), was incorporated into the CMCht/PAMAM and administered directly into the CSF in the *cisterna magna* of Wistar rats. This study showed that MP-loaded CMCht/PAMAM broadly diffused in the healthy rat brain following administration in the CSF, while delivering MP. The incorporation of MP into a dendrimer formulation was responsible for modulation of the metabolic activity of microglia.^43^ Recently, a few studies have reported on another approach using magnetic nanoparticles (MNPs) for magnetic guidance of drugs and their targeted delivery.^44,45^ Lueshen et al.^46^ developed a physiologically and anatomically consistent in vitro human spine model that reproduced natural CSF pulsations to infused gold-coated magnetite nanoparticles. This system allowed a more targeted drug delivery to specific regions using an external magnetic field. The same authors proved the increase in targeting efficacy using magnetizable implants.^47^ More recently, they validated this intrathecal magnetic drug targeting (IT-MDT) approach in vivo using Sprague-Dawley rats.^48^

With regard to the linear polymers, one of the most widely used for drug delivery is poly(d,l-lactic-co-glycolic acid) (PLGA), since it is biodegradable and there is minimal systemic toxicity associated with CNS applications.^49^ Minocycline-loaded PLGA nanoparticles showed no in vitro degradation of the drug in artificial CSF during the seven-day stability study.^50^

In addition to the use of nanoparticles for drug or growth factor delivery systems, nanoparticles have gained special attention for stem cell tracking, namely, MNPs for detection by MRI. Cell tracking is important, as it allows for a better understanding and optimization of cell-mediated effects.^51^ Meta-analyses of cell-mediated effects in preclinical cell therapy studies in neurological disorders have revealed the primary therapeutic mechanisms^52^ and nanotechnology can offer a non-invasive monitoring system for further improving these positive effects.

## Non-invasive imaging of intrathecally injected biomaterials

**Fig. 4:**
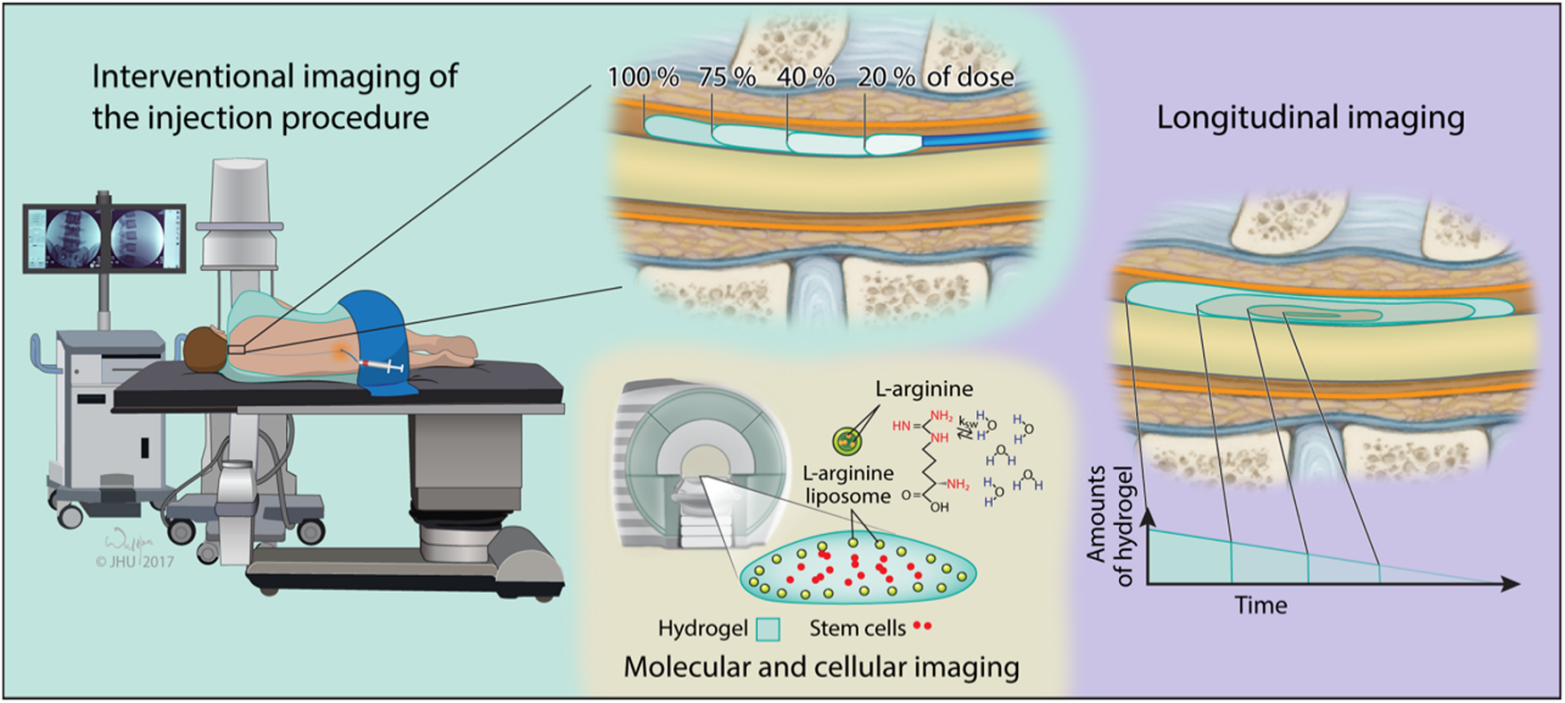
Non-invasive imaging of intrathecally injected cell/biomaterial constructs

The advantages of injecting cells via intrathecal route are clear as discussed above. The challenge, however, is related to the uncertainty about cell biodistribution and potentially high variability of biomaterial/cell dispersal. While direct intraparenchymal injection results in highly reproducible and rather predictable placement,^53^ in case of injection into the fluid compartments uncertainty is high. This uncertainty is related to the mixing and dilution of biomaterial with CSF and its redistribution with fluid circulation. In this context, it is highly desirable to monitor injected biomaterials using non-invasive imaging. Even though literature on image-guided intrathecal injection of biomaterial/cell composites is very limited, application of this approach in other organ systems is quite abundant thus here we will focus on the rationale and the needs of imaging for intrathecal injection, and, will suggest imaging strategies that are best suited to fulfill these needs. There are three areas where imaging could assist in improving the success of intrathecal biomaterial/cell injection including monitoring the procedure of biomaterial injection in real-time, longitudinal assessment and finally, molecular imaging of the local microenvironment at the injection site (Fig. 4).

Imaging is expected to provide a breadth of information about the biomaterial itself, the embedded cells, and the graft-tissue interactions in the context of the healing process. Moreover, it is important that imaging requirements, such as incorporation of the contrast agent, does not alter the properties of the biomaterial, does not interfere with biology of embedded cells, and, ideally, does not compromise functional assessment by any imaging modalities.^54^ The selection of the optimal imaging technique for a particular application is not a trivial task, and multiple factors have to be considered. One example is the case of a superficial injection within a few millimeters under the skin, in which a low-energy imaging method (e.g., photoacoustic, fluorescent or bioluminescent) is sufficient to track hydrogel integrity and release of therapeutics within the injected organism.^55,56^ However, to monitor delivery into deep structures, such as intravascular or intra-organ injection, including the intrathecal space, tomographic techniques (e.g., MRI, PET, and X-ray) are usually required.^57–59^ Each imaging modality has unique properties with advantages and disadvantages; here, we will review these features as they relate to placement of biomaterials in the intrathecal space.

### Interventional imaging of the injection procedure

Placement of the intrathecal catheter at the desired level of the spinal cord is performed under fluoroscopic visualization and this procedure is routinely performed clinically with an example of baclofen pump implantation for treatment of dystonia.^60^ Infusion of drugs as fluid suspension can be performed without complications even chronically over weeks or months, as they simply mix with the CSF. Implantation of hydrogels, however, is more demanding and requires more careful approach as it is desired that the material would persist locally in a specific and selected area of the intrathecal space over the required period of time. When biomaterial is being injected, dynamic imaging can be used to assure the precision of targeting and to minimize the risk of excessive injection or misplacement. Indeed, misplacement of the biomaterial may have negative consequences beyond the suboptimal therapeutic effect, as misinjected graft could lead to unwanted adverse effects. Infusion into intrathecal space results in its rostral or caudal spread from the catheter tip, or combination of the two. Without imaging in real-time, proper placement is practically impossible. Monitoring biomaterial biodistribution can be accomplished with the use of X-ray. X-ray imaging based on phase contrast proved to be capable of detecting the hydrogel structure without the addition of contrast agent. The nonporous and porous PEG hydrogels were discernible from surrounding water or soft tissue in vitro without the use of contrast agents.^61^ In addition, Faraj et al.^62^ demonstrated that high-resolution computed tomography (micro-CT) enables the determination of the structure of soft scaffolds in vitro. In addition to low X-ray attenuation of collagen-based hydrogels, different combinations of contrast agents were used. It was shown that the application of osmium tetroxide and uranyl acetate, or a combination of uranyl acetate and lead citrate, allowed high-resolution 3D imaging of the structure of the scaffolds. Lei et al.^63^ showed the possibility of non-invasive tracking of radiopaque thermo-reversible hydrogels after implantation, and the opportunity to obtain detailed 3D morphological information in a real-time manner. Due to its accessibility and low cost, X-ray imaging techniques are promising tools for hydrogel observation but ionizing radiation is a concern and the motivation for developing other imaging modalities such as MRI. Indeed MRI is extensively used in biomedicine^2,64^ and recently significant progress has been made in interventional MRI including real-time assessment of injected cells^65^ and biomaterials.^66^ MRI has many features that make it one of the most desirable and preferable imaging modalities for imaging in biomedicine. These features include excellent soft tissue contrast, tomographic capabilities, excellent anatomical information, and it is non-invasive and radiation-free. MRI has been widely used for in vivo and ex vivo analysis of biomaterials and transplanted cells, and, while detection of native components of hydrogel has been demonstrated,^67^ labeling with MRI contrast agent is usually required. Imaging moiety is often introduced in the form of a nanoparticle such as iron oxide^68^ gadolinium^69,70^ or fluorine nanoemulsions.^71^ Various types of nanocarriers have been developed including more advanced systems with both therapeutic and diagnostic components (Theranostic agents). Good example of such theranostic agents in the contex of regenerative medicine are iron oxide-based particles loaded with microRNAs for simultaneous tracking and manipulation of transplanted cells.^72^ Another application of nanoparticles for imaging is by combining contrast mechanism for more than one modality known as multimodality nanoparticles, such as a combination of PET, near-infrared fluorescence, and MRI^73^ While there are no published reports on intrathecal injection of biomaterials guided by dynamic imaging we would like to review several relevant applications based on imaging of labeled stem cells.

Superparamagnetic iron oxide nanoparticles (SPION) are of particular interest for stem cell tracking, since they, in general, do not result in adverse effects on cell survival and functionality in terms of differentiation capacity, gene expression profiles, or migratory capacity.^8,74^ Bigini et al.^75^ labeled human fetal cells with SPIO nanoparticles in the brain of mice with motor neuron disease. It was reported that transplanted cells rapidly diffused throughout the fourth ventricle at the level of the spinal cord, and the labeling did not affect the proliferation and metabolic activity of cells.^75^ MRI has been successfully used for the dynamic assessment of cell biodistribution in real-time during intra-arterial injection in rodents^76^ where imaging was instrumental in optimizing the procedure of cell delivery. Recently, real-time MRI monitoring has been applied to predict and improve the precision of cell delivery into the brain in small-animal and large-animal models.^2^ While real-time assessment of intrathecal injection of the biomaterial with MRI has not been reported it is highly feasible and application of this technique would likely provide important clues on how to assure reproducibility, reliability and efficacy of intrathecal biomaterial/cell delivery.

### Longitudinal imaging

While most of the requirements for imaging of biomaterials over time are common with described above real-time imaging, there are few unique challenges related to specificity. The advantage of interventional imaging is an access to pre-injection baseline and unequivocal interpretation of post injection images following simple subtraction process. Longitudinal imaging introduces uncertainty as some pathological processes may imitate signal features of the graft with an example of hypointense signal produced by both iron oxide nanoparticle-based contrast agents and a hemorrhage.^77^ Nevertheless both X-ray and MRI has been used for monitoring injected biomaterials over time. One example is a study by Appel et al.^78^ showing that X-ray phase contrast (XPC) CT application allows 3D visualization and quantification of hydrated soft tissues and PEG hydrogels in vivo, with no contrast agents. The XPC CT imaging enabled a clear distinction between surrounding tissue after transplantation and the hydrogel structure. Also, tantalum, known for its biological properties of facilitating soft tissue regeneration and vascularization, is, at the same time a promising contrast agent for X-ray imaging, enhancing the visibility of cells and biomaterial grafts.^79^ MRI has been shown instrumental in the assessment of biomaterials biodegradability in a study reported by Yang et al..^80^ That work showed that the biodegradability level of hydrogels can be monitored and quantified in vivo using an alteration of ^19^F intensity. Recently, MR imaging of hydrogels transplanted as scaffolds, as well as an injectable formulation, has been reported.^81^ Zhang et al.^82^ presented in situ, cross-linkable, HA-based hydrogels, hybridized with iron oxide nanoparticles to enable their detection on MRI. Another interesting study used iron oxide as a contrast agent and multiple crystals of iron oxide were encapsulated inside the polyacrylamide matrix, yielding very high relaxivity. Because of the monitoring of the amount of liberated iron oxide nanoparticles by the hyaluronidases, it was possible to monitor and analyze the hydrogel degradation.^83^ Bible and co-workers^84^ showed non-invasive imaging of the ECM scaffold in a stroke-damaged rat brain using ^19^F MRI, demonstrating that transplantation of neural stem cells embedded in xenogeneic ECM scaffolds resulted in uniformly distributed cells throughout the lesion cavity.

### Molecular and cellular imaging of the local microenvironment

One of the most challenging but highly rewarding tasks is non-invasive imaging of the local microenvironment reporting on the molecular processes within and around the injected biomaterial. Greatest potential in that area has recently developed molecular MRI technique that is based on chemical exchange saturation transfer (CEST-MRI), which is a novel molecular MRI technique with the important advantage that components which naturally occur in the biomaterials are used to generate contrast.^85^ Endogenous molecules dependent on the content of labile protons in the sample may generate CEST contrast, thus facilitating their detection by CEST-MRI without any additional contrast agent. Liang et al.^67^ reported on the use of CEST-MRI to monitor biodegradation of a gelatin-containing HA-based hydrogels, both in vitro as well as in vivo in mice. In addition, Jin et al.^86^ described the use of CEST for both in vitro and in vivo examination of ECM hydrogels in a rat stroke model. In vitro CEST imaging was instrumental in demonstrating dynamic changes in the different components of the ECM inside the hydrogel. Moreover, in vivo CEST examination allowed detection of ECM hydrogel distribution and degradation that strongly corresponded to histological studies post mortem.^86^ Chan et al.^57^ described pH nanosensor-based magnetic resonance imaging as a modality with which to image encapsulated cell death in vivo. Ultrapure, low-viscosity, high-guluronate alginate and ultrapure, low-viscosity, high-mannuronate alginate (NovaMatrix), and liposomes containing l-arginine, were used for microcapsule preparation (Fig. 4). It was shown that LipoCEST nanosensors were sensitive enough to detect cell death caused by incomplete immunoprotection.

In the context of monitoring local cellular microenvironment it is important to mention bioluminescence imaging (BLI). This technique is based on a reporter gene (e.g., firefly luciferase) that is expressed by the cells of interest. As the photon signal is generated only by metabolically active cells and is dependent on access to ATP, it is an excellent method for in vivo monitoring of cell viability. Even though this modality is only applicable to small-animal models it is invaluable for assessment of biomaterial/cell composites. As described by Allen et al.^87^ *GFP/Luc* hMSCs were embedded in (low-molecular-weight-irradiated RGD-functionalized) alginate or in (SeaPlaque) agarose hydrogels and injected subcutaneously in rats. Although both hydrogel types showed a linear correlation between the BLI signal and the live cell number using a 30-min imaging protocol, there was a difference in the magnitude of the BLI signal measured between the agarose and alginate materials, exemplifying the utility of BLI for monitoring interactions between biomaterials and embedded cells. In addition, Liang et al. showed that BLI is useful for monitoring the viability of cells embedded in injectable hydrogel.^88^ That study showed that HA-based hydrogels improved the survival and proliferation of three different transplanted cell lines (C17.2 neural stem cells, ReNcells, and glial progenitors). Similarly, Levit et al. used Luc hMSCs encapsulated in a low-viscosity guluronic acid-based alginate (LVG, Novamatrix) in a rat myocardial infarction model.^89^ BLI showed that encapsulation of MSCs in an LVG hydrogel minimized scar formation and improved cardiac function. Similarly, luciferase-expressing ASCs (*Luc* ASCs) embedded within fibrin scaffolds and transplanted in the injured heart were tracked with BLI as reported by Yang et al.^90^

Overall, several imaging modalities offer unique opportunities for non-invasive monitoring of the intrathecal injection. Each of the modalities has strengths and limitations with multimodality approach likely required for providing most comprehensive information.

## Concluding remarks and future trends

The spinal cord is clearly one of the most difficult targets for the transplantation of stem cells and one of the most challenging elements is the route of cell delivery. Diseases characterized by disseminated pathology require global cell engraftment, and, in these cases, intrathecal injection seems to be highly recommended. The infusion of cell suspensions directly into the CSF can result in cell sedimentation and suboptimal cell biodistribution, and hence, the need for supporting biomaterials. The transplantation of cells embedded in injectable hydrogels addresses the problem of possible cell sedimentation while mimicking the native extracellular matrix, additional benefits of hydrogels include bioadhesiveness and offer the potential for supplementation with nanocarriers laden with molecules that can promote survival and differentiation of stem cells. Finally, imaging technology offers unique opportunities for the characterization of cell-biomaterial composites both in vitro and after transplantation.

As shown above, there have been several reports that advocate the use of the intrathecal route for stem cell delivery, and progress in the field of biomaterials is unprecedented, offering a breadth of injectable hydrogel materials that meet the requirements of intrathecal injection. While the applications for hydrogel-embedded stem cells for intrathecal transplantation are limited, we certainly hope this review will spark more interest in this exciting area. Recent progress in the non-invasive imaging of biomaterials, and, particularly, clinically applicable modalities, such as MRI, will help guide the development of more effective and safer protocols for intrathecal therapeutic cell transplantation.
